# Drug-based pain management in people with dementia after hip or pelvic fractures: a systematic review protocol

**DOI:** 10.1186/s13643-016-0296-3

**Published:** 2016-07-13

**Authors:** S. Kuske, K. Moschinski, S. Andrich, A. Stephan, I. Gnass, E. Sirsch, A. Icks

**Affiliations:** Institute for Health Services Research and Health Economics, Faculty of Medicine, Economics, Heinrich-Heine-University, Moorenstraße 5, 40225 Düsseldorf, Germany; Paul Langerhans Group for Health Services Research and Health Economics, German Diabetes Center (DDZ), Leibniz Institute for Diabetes Research, Heinrich-Heine-University, Auf’m Hennekamp 65, 40225 Düsseldorf, Germany; German Centre for Diabetes Research (DZD), Ingolstädter Landstraße 1, 85764 Neuherberg, Germany; Institute of Nursing Science and Education, Paracelsus Medicine University, Salzburg, Austria; Faculty of Nursing Science, Vallendar Philisophic-Theological College, Palottistraße 3, 56179 Vallendar, Germany; Fliedner Fachhochschule, University of Applied Sciences, Geschwister-Aufricht-Straße 9, 40489 Düsseldorf, Germany; Department of Nursing, RWTH Aachen University Hospital, 52074 Aachen, Germany

**Keywords:** Pain management, Analgesics, Drugs, Hip fractures, Pelvic fractures, Dementia, Alzheimer, Cognitive impairment, Cognitive disorders

## Abstract

**Background:**

Studies show that people with dementia do not receive the same amount of analgesia after a hip or pelvic fracture compared to those without cognitive impairment. However, there is no systematic review that shows to what extent and how drug-based pain management is performed for people with dementia following a hip or pelvic fracture. The aim of this systematic review is to identify studies addressing drug-based pain management for people with dementia who have had a hip or pelvic fracture for which they had either an operation or conservative treatment. We will analyse to what extent and how the drug-based pain treatment for people with dementia is performed across all settings and how it is assessed in the studies.

**Methods:**

The development of this systematic review protocol was guided by the PRISMA-P requirements, which will be taken into consideration during the review procedure. MEDLINE, EMBASE, CINAHL, Web of Knowledge and ScienceDirect will be searched, using keywords such as “analgesia”, “dementia”, “cognitive impairment”, “pain treatment”, “hip fracture” or “pelvic fracture”. Publications published up to January 2016 will be included. The data extraction and a content analysis will be carried out systematically, followed by a critical appraisal.

**Discussion:**

This review will provide a valuable overview on the current evidence on drug-based pain management for PwD in all settings who were conservatively treated after a hip or pelvic fracture. The review may expose a need to enhance pain management for PwD. It may also provide motivation for healthcare providers and policymakers to give this topic their attention and to facilitate further research by considering aspects of care transitions in all settings.

**Systematic review registration:**

PROSPERO CRD42016037309

**Electronic supplementary material:**

The online version of this article (doi:10.1186/s13643-016-0296-3) contains supplementary material, which is available to authorized users.

## Background

Hip and pelvic fractures represent 13.3 % of all fractures [1]. It is expected that the absolute number of hip fractures will rise worldwide from 1.7 million in 1990 to 6.3 million in 2050 [2], although age-standardised risks of having hip fractures are levelling off or are decreasing, especially in Western countries [3, 4]. In contrast, age-standardised risks for pelvic fractures have increased over the last decades [5].

Hip and pelvic fractures are often a consequence of falls [5, 6]. A common reason for fall-related fractures among older people seems to be dementia [7]. In August 2015, an estimated number of 46.8 million people worldwide suffered from dementia [8] and this number is expected to double every 20 years in the future [9].

The incidence of dementia increases significantly, comparable to the incidence of hip and pelvic fractures, beginning at the age of 60 [8]. The risk of experiencing a fall-related fracture is two to three times higher for people with dementia (PwD) than for cognitively intact persons. The risk of falling ranges per annum between 60 and 80 %. Impaired vision, balance problems and reduced muscular strength are seen as consequences of dementia that cause a risk of falling for PwD [10].

The pain management after a fracture is crucial for PwD [11]. Morrison and Siu [12] showed that a majority of the participants diagnosed with dementia who had previously experienced a hip fracture received only one third of the analgesic drugs that are given to cognitively intact patients. Studies have pointed out that PwD, in comparison to those without dementia, are often not able to verbalise the pain [13, 14] and need more attention due to an increased risk of poorer postoperative outcomes [15] and an increased sensibility of pain [16]. Furthermore, severe pain raises the risk of delirium in cognitively impaired individuals [17]. However, a systematic review is missing that shows to what extent and how the drug-based pain management is performed for people with dementia following a hip or pelvic fracture. The aim of the systematic review is, therefore, to identify studies addressing drug-based pain management for people with dementia who have had a hip or pelvic fracture for which they had either an operation or conservative treatment. We will analyse to what extent and how drug-based pain treatment is performed in all settings and how it is assessed in the studies.

## Method/design

This systematic review will be performed in line with the quality requirements of the PRISMA-P guideline [18]. The PRISMA-P file is included for more details (see Additional file 1). MEDLINE, EMBASE, CINAHL, Web of Knowledge and ScienceDirect will be searched. Studies published up to January 2016 will be included. Titles and abstracts have to be in German or English. Backward citation tracking and forward citation tracking of selected literature will be added [19].

The search strategy will be set up by using the database-specific vocabularies (MeSH, EMTREE) and additional free text terms. The preliminary search algorithm is shown in Tables 1 and 2. The aim is to identify all relevant publications and at the same time to identify only those publications that are relevant (i.e. high sensitivity, low specificity), thus yielding a low number-needed-to-read (NNR) and minimising the subsequent workload. The search algorithm will be counter-checked by experienced reviewers and then piloted. Included search terms will be, for example, “analgesia”, “dementia”, “cognitive impairment”, “pain treatment”, “hip fracture” or “pelvic fracture”.

### Inclusion and exclusion criteria

Literature addressing drug-based pain management for PwD following hip or pelvic fractures in all settings will be included. Original articles reporting qualitative, quantitative or mixed methods studies and grey literature, such as dissertations, will be included. Letters, short reports, abstracts, editorials, comments or discussion papers will be screened in order to identify further original studies. We will include publications in which our topic has a primary or secondary focus. Publications without available references will be excluded.

### Study selection process

Inclusion and exclusion criteria will be pre-tested by conducting a comprehensive pre-screening and by discussing first core publications. Articles will be selected by title and by screening abstracts and will be checked by all the reviewers independently. The full texts selected will be double-checked. Unclear decisions will be resolved by consulting an additional reviewer.

**Table 1:** Search terms

Topic	Mesh terms	Search terms
Dementia	Dementia[Mesh]; Frontotemporal Dementia[Mesh]; AIDS Dementia Complex[Mesh]; Alzheimer’s[Mesh]; Delirium, Dementia, Amnestic, Cognitive Disorders[Mesh]; Lewy Body Disease[Mesh]; Cognitive impairment, mild[MeSH Terms]; Dementia, multi-infarct[Mesh]	vascular dementia; alzheimer*; alzheimer disease; dementia; dement*; Cognit* AND (disord* OR impair* OR declin* OR function*)
Elderly	Frail elderly[MeSH Terms]; Aged[MeSH Terms]; Aged, 80 and over[MeSH Terms]	elderly; elders; older adults
Pain Management	Pain management[MeSH Terms]; Narcotics[MeSH Terms]; Analgesics[MeSH Terms]	pain treatment; pain treat*; pain therapy; pain therap*; analgesic; analgesia; pain AND (medication OR medicat* OR drugs OR drug OR prescribing OR prescrib*); ((medicat*) OR (medication) OR (drug) OR (drugs) OR (prescrib*)) AND (pain)
Hip fracture	Femoral neck fractures[MeSH Terms]; Hip fractures[MeSH Terms]	femoral neck fracture; femoral neck fract*; femur neck fracture; femur neck fract*; femoral neck trauma; femur neck trauma; trochanteric fracture; trochant* fract*; trochanteric trauma; intertrochanteric fracture; intertrochant* fract*; subtrochanteric fracture; subtrochant* fract*; proximal femur fracture; proximal femur fract*; proximal femur trauma; proximal femoral fracture; proximal femoral fract*; proximal femoral trauma
Pelvic fracture	Pelvic bones[MeSH Terms] AND (fracture OR fract* OR trauma OR traumato*)	pelvic fracture; pelvic fract*; pelvic-fract*; fract* pelvis; pelv* fract*; pelv* trauma; pelvic traumato*; (acetabulum OR ilium OR ischium OR pubic bone) AND (fracture OR fract* OR trauma OR traumato*)

**Table 2:** Search queries

#	MEDLINE via PUBMED
1	((dementia[Mesh]) OR Frontotemporal Dementia[Mesh]) OR AIDS Dementia Complex[Mesh]) OR Alzheimer’s[Mesh]) OR Delirium, Dementia, Amnestic, Cognitive Disorders[Mesh]) OR Lewy Body Disease[Mesh]) OR vascular dementia) OR cognitive impairment, mild[MeSH Terms]) OR alzheimer*) OR alzheimer disease) OR (dementia OR dement*)) OR dementia, multi-infarct[Mesh]) OR (Cognit* AND (disord* OR impair* OR declin* OR function*))))
2	frail elderly[MeSH Terms] OR aged[MeSH Terms] OR aged, 80 and over[MeSH Terms] OR elderly OR elders OR older adults
3	#1 OR #2
4	(((((pain management[MeSH Terms]) OR Pain OR (((pain treat* OR pain treatment OR pain therapy OR pain therap* OR narcotics[MeSH Terms] OR analgesics[MeSH Terms] OR analgesics, opioid[MeSH Terms] OR analgesic OR analgesia OR analge*))))) OR (((Pain) AND (medication OR medicat* OR drugs OR drug OR prescribing OR prescrib*))))
5	(((((((((pelvic fracture OR pelvic fract* OR pelvic-fract* OR (fract* pelvis) OR pelv* fract* OR pelv* trauma OR pelvic traumato*))) OR ((pelvic bones[MeSH Terms]) AND ((fracture OR fract* OR trauma OR traumato*)))) OR ((((acetabulum OR ilium OR ischium OR pubic bone) AND (fracture OR fract* OR trauma OR traumato*))))))) OR (((femoral neck fracture OR femoral neck fract* OR femur neck fracture OR femur neck fract* OR femoral neck trauma OR femur neck trauma OR trochanteric fracture OR trochant* fract* OR trochanteric trauma OR intertrochanteric fracture OR intertrochant* fract* OR subtrochanteric fracture OR subtrochant* fract*)) OR (proximal femur fracture OR proximal femur fract* OR proximal femur trauma OR proximal femoral fracture OR proximal femoral fract* OR proximal femoral trauma)) OR ((femoral neck fractures[MeSH Terms] OR hip fractures[MeSH Terms]))))))))
6	#3 AND #4 AND #5
	(((((((((dementia[Mesh]) OR Frontotemporal Dementia[Mesh]) OR AIDS Dementia Complex[Mesh]) OR Alzheimer’s[Mesh]) OR Delirium, Dementia, Amnestic, Cognitive Disorders[Mesh]) OR Lewy Body Disease[Mesh]) OR vascular dementia) OR cognitive impairment, mild[MeSH Terms]) OR alzheimer*) OR alzheimer disease) OR (dementia OR dement*)) OR dementia, multi-infarct[Mesh]) OR (Cognit* AND (disord* OR impair* OR declin* OR function*))))))) OR ((frail elderly[MeSH Terms] OR aged[MeSH Terms] OR aged, 80 and over[MeSH Terms] OR elderly OR elders OR older adults))))) AND (((((((pain management[MeSH Terms]) OR Pain OR (((pain treat* OR pain treatment OR pain therapy OR pain therap* OR narcotics[MeSH Terms] OR analgesics[MeSH Terms] OR analgesics, opioid[MeSH Terms] OR analgesic OR analgesia OR analge*))))) OR (((Pain) AND (medication OR medicat* OR drugs OR drug OR prescribing OR prescrib*))))))) AND ((((((((((pelvic fracture OR pelvic fract* OR pelvic-fract* OR (fract* pelvis) OR pelv* fract* OR pelv* trauma OR pelvic traumato*))) OR ((pelvic bones[MeSH Terms]) AND ((fracture OR fract* OR trauma OR traumato*)))) OR ((((acetabulum OR ilium OR ischium OR pubic bone) AND (fracture OR fract* OR trauma OR traumato*))))))) OR (((femoral neck fracture OR femoral neck fract* OR femur neck fracture OR femur neck fract* OR femoral neck trauma OR femur neck trauma OR trochanteric fracture OR trochant* fract* OR trochanteric trauma OR intertrochanteric fracture OR intertrochant* fract* OR subtrochanteric fracture OR subtrochant* fract*)) OR (proximal femur fracture OR proximal femur fract* OR proximal femur trauma OR proximal femoral fracture OR proximal femoral fract* OR proximal femoral trauma)) OR ((femoral neck fractures[MeSH Terms] OR hip fractures[MeSH Terms])))))))))
*#*	*CINAHL via EBESCO*
1	“((MH “Dementia+”) OR “dementia” OR (MH “Frontotemporal Dementia+”) OR (MH “Dementia, Vascular+”) OR (MH “Delirium, Dementia, Amnestic, Cognitive Disorders+”) OR (MH “Dementia, Multi-Infarct”) OR (MH “AIDS Dementia Complex”) OR (MH “Lewy Body Disease”) OR (MH “Dementia, Senile+”) OR (MH “Dementia, Presenile+”)) OR (Delirium OR Dementia OR Amnestic OR vascular dementia OR cognitive impairment OR alzheimer* OR alzheimer disease OR dementia OR dement* OR (Cognit* AND (disord* OR impair* OR declin* OR function*))
2	“((MH “Frail Elderly”) OR “elderly” OR (MH “Aged, 80 and Over”) OR (MH “Aged, Hospitalized”) OR (MH “Health Services for the Aged”) OR (MH “Aged+”)) OR (elderly OR aged OR elders OR older adults)
3	#1 OR #2
4	((MH “Pain+”) OR “pain” OR (MH “Pelvic Pain+”) OR (MH “Pain Clinics”) OR (MH “Pain Measurement”) OR (MH “Treatment Related Pain”) OR (MH “Postoperative Pain”) OR (MH “Back Pain+”)) OR (pain OR pain treat* OR pain treatment OR pain therapy OR pain therap* OR analgesics OR analgesic OR analgesia OR analge* OR (((Pain) AND (medication OR medicat* OR drugs OR drug OR prescribing OR prescrib*)))))
5	(MH “Spinal Fractures+”) OR (MH “Sacral Fractures, Stress”) OR (MH “Femoral Fractures+”) OR (MH “Hip Fractures, Stress”) OR (MH “Fractures, Ununited+”) OR (MH “Pelvic Fractures”) OR (MH “Fractures, Stress+”) OR (MH “Fractures, Open”) OR (MH “Humeral Fractures+”) OR (MH “Fractures, Compression+”) OR (MH “Fractures, Comminuted”) OR (MH “Fractures, Closed”) OR (MH “Hip Fractures+”) OR (MH “Fractures+”) OR (MH “Fracture Healing”) OR “fracture”) OR (pelvic fracture OR pelvic fract* OR pelvic-fract* OR fract* pelvis OR pelv* fract* OR pelv* trauma OR pelvic traumato* OR ((pelvic bones[MeSH Terms]) AND ((fracture OR fract* OR trauma OR traumato*)))) OR ((acetabulum OR ilium OR ischium OR pubic bone) AND (fracture OR fract* OR trauma OR traumato* OR femoral neck fracture OR femoral neck fract* OR femur neck fracture OR femur neck fract* OR femoral neck trauma OR femur neck trauma OR trochanteric fracture OR trochant* fract* OR trochanteric trauma OR intertrochanteric fracture OR intertrochant* fract* OR subtrochanteric fracture OR subtrochant* fract* OR proximal femur fracture OR proximal femur fract* OR proximal femur trauma OR proximal femoral fracture OR proximal femoral fract* OR proximal femoral trauma))
6	#3 AND #4 AND #5
*#*	*EMPBASE via SCOPUS*
1	((Cognit* AND (disord* OR impair* OR declin* OR function*))) OR (dementia OR Frontotemporal Dementia OR AIDS Dementia Complex OR Alzheimer’s OR Lewy Body Disease OR vascular dementia OR mild cognitive impairment OR alzheimer* OR alzheimer disease OR dementia OR dement* OR multi-infarct dementia)
2	frail elderly OR aged OR elderly OR elders OR older adults
3	#1 OR #2
4	(((medicat*) OR (medication) OR (drug) OR (drugs) OR (prescrib*)) AND (Pain)) OR (pain treat* OR pain treatment OR pain therapy OR pain therap* OR Narcotics OR Analgesics OR analgesic OR analgesia OR analge*)
5	((pelvic bones OR acetabulum OR ilium OR ischium OR pubic bone) AND (fracture OR fract* OR trauma OR traumato*)) OR (pelvic fracture OR pelvic fract* OR pelvic-fract* OR fract* pelvis OR pelv* fract* OR pelv* trauma OR pelvic traumato*)
6	(femoral neck OR femoral neck OR femur neck OR trochanteric OR trochant* OR intertrochanteric OR intertrochant* OR subtrochanteric OR subtrochant* OR proximal femur OR proximal femoral OR hip) AND (fracture OR fract* OR trauma OR traumato*)
7	#5 OR #6
8	#3 AND #4 AND #7
*#*	*SCIENCEDIRECT*
1	((Cognit* AND (disord* OR impair* OR declin* OR function*))) OR (dementia OR Frontotemporal Dementia OR AIDS Dementia Complex OR Alzheimer’s OR Lewy Body Disease OR vascular dementia OR mild cognitive impairment OR alzheimer* OR alzheimer disease OR dementia OR dement* OR multi-infarct dementia)
2	frail elderly OR aged OR elderly OR elders OR older adults
3	#1 OR #2
4	(((medicat*) OR (medication) OR (drug) OR (drugs) OR (prescrib*)) AND (Pain)) OR (pain treat* OR pain treatment OR pain therapy OR pain therap* OR Narcotics OR Analgesics OR analgesic OR analgesia OR analge*)
5	((pelvic bones OR acetabulum OR ilium OR ischium OR pubic bone) AND (fracture OR fract* OR trauma OR traumato*)) OR (pelvic fracture OR pelvic fract* OR pelvic-fract* OR fract* pelvis OR pelv* fract* OR pelv* trauma OR pelvic traumato*)
6	(femoral neck OR femoral neck OR femur neck OR trochanteric OR trochant* OR intertrochanteric OR intertrochant* OR subtrochanteric OR subtrochant* OR proximal femur OR proximal femoral OR hip) AND (fracture OR fract* OR trauma OR traumato*)
7	#5 OR #6
8	#3 AND #4 AND #7
	(((medicat*) OR (medication) OR (drug) OR (drugs) OR (prescrib*)) AND (Pain)) AND ((((pelvic bones OR acetabulum OR ilium OR ischium OR pubic bone) AND (fracture OR fract* OR trauma OR traumato*)) OR (pelvic fracture OR pelvic fract* OR pelvic-fract* OR fract* pelvis OR pelv* fract* OR pelv* trauma OR pelvic traumato*)) OR ((femoral neck OR femoral neck OR femur neck OR trochanteric OR trochant* OR intertrochanteric OR intertrochant* OR subtrochanteric OR subtrochant* OR proximal femur OR proximal femoral OR hip) AND (fracture OR fract* OR trauma OR traumato*))) AND (((Cognit* AND (disord* OR impair* OR declin* OR function*))) OR (dementia OR Frontotemporal Dementia OR AIDS Dementia Complex OR Alzheimer’s OR Lewy Body Disease OR vascular dementia OR mild cognitive impairment OR alzheimer* OR alzheimer disease OR dementia OR dement* OR multi-infarct dementia))
*#*	*WEB OF KNOWLEDGE*
1	Dementia (15)
2	Elderly (17)
3	Dementia OR Elderly (23)
4	Pain Management (22)
5	Pelvic fracture (28)
6	Hip Fracture (29)
7	Hip Fracture OR Pelvic Fracture (30)
8	Dementia AND Pain Management AND #30 (31)
9	(Dementia OR Elderly) AND Pain Management AND #30 (32)
10	#9 Englisch OR German

Inter-rater reliability will be determined following title and abstract screening as well as after reviewing the full texts.

### Data extraction and synthesis

A data extraction sheet will be developed according to Cochrane requirements [20]. First, an overview of the studies will be set up, stating author, year of publication, study design, settings, study objectives, findings regarding the extent of and methods used in the drug-based pain treatment of PwD in all settings and the results of the critical appraisal. Second, data will be extracted and analysed with regard to the study methods (e.g. data collection by chart review or questionnaire), outcomes measures, type of data (e.g. clinical or administrative data), sample size, comparison between PwD and cognitively intact people, participant subgroups and predictors. Third, data will be extracted and analysed that addresses the assessments of the studies identified, e.g. mental tests, pain scales and type of medication, in line with the World Health Organization classification. A coding protocol will be used.

After data extraction, a content analysis [21] will be conducted by developing categories (deductively and inductively) according to the topics of the review questions. We will also perform a descriptive quantitative analysis, taking for example prevalences or odds ratios into consideration.

The analysis and synthesis of analysing differences and similarities between the studies will be performed as a peer group process.

### Critical appraisal and risk of bias

All the instruments used for the critical appraisal will take the risk of bias in the studies into account. Each type of study will be assessed separately. The quality assessment of the studies will be done by using the instruments of the Scottish Intercollegiate Guideline Networks (SIGN). These instruments offer a broad spectrum of checklists for the different study types, give clear and easily understandable criteria and provide solid results [22]. Studies that are not addressed in the SIGN guideline will be analysed using the critical appraisal tools of the National Institute for Health and Care Excellence (NICE) [23]. Mixed methods studies, which are addressed neither by the SIGN nor by the NICE guidelines, will be assessed with the Mixed Methods Appraisal Tool (MMAT) [24, 25]. This process will be performed independently by two reviewers.

## Discussion

This review will provide a valuable overview on the current evidence of drug-based pain management for PwD in all settings who have had a hip or pelvic fracture which was treated either conservatively or by operating. The review results can be used to show if there is a need to improve pain management for PwD and can provide recommendations regarding the monitoring of appropriate medication treatment given to PwD. The systematic review can motivate healthcare providers and policymakers to give their attention to this topic. Finally, this review can be a basis for further research by considering aspects of care transitions across all settings.

## Abbreviations

AI, Andrea Icks; AS, Astrid Stephan; ES, Erika Sirsch; IG, Irmela Gnass; KM, Kai Moschinski; PwD, People with Dementia; SA, Silke Andrich; SK, Silke Kuske

## Additional file

Additional file 1: PRISMA-P (Preferred Reporting Items for Systematic review and Meta-Analysis Protocols) 2015 checklist: recommended items to address in a systematic review protocol*. (DOC 96 kb)
