# Supplementary material for: Drug-based pain management in people with dementia after hip or pelvic fractures: a systematic review protocol
Source: Syst Rev. 2016 Jul 13;5:113. doi: 10.1186/s13643-016-0296-3 (PMC4944510; doi:10.1186/s13643-016-0296-3)
Supplement: Additional file 1: — PRISMA-P (Preferred Reporting Items for Systematic review and Meta-Analysis Protocols) 2015 checklist: recommended items to address in a systematic review protocol*. (DOC 96 kb) [file 13643_2016_296_MOESM1_ESM.doc]

**ADDITIONAL FILE 1**

**PRISMA-P (Preferred Reporting Items for Systematic review and Meta-Analysis Protocols) 2015 checklist: recommended items to address in a systematic review protocol***

| Section and topic | Item No | Checklist item |
| --- | --- | --- |
| ADMINISTRATIVE INFORMATION | | |
| **Title:** |  |  |
| Drug-based pain management in people with dementia after hip or pelvic fractures: a systematic review protocol | 1a | Identify the report as a protocol of a systematic review |
| This review is not an update. | 1b | If the protocol is for an update of a previous systematic review, identify as such |
| **Registration:**  In accordance with the guidelines, our systematic review protocol was registered with the International Prospective Register of Systematic Reviews (PROSPERO) on 11. April 2016 (CRD42016037309) | 2 | If registered, provide the name of the registry (such as PROSPERO) and registration number |
| **Authors:**  Kuske S, Moschinski K, Andrich S, Stephan A, Gnass I, Sirsch E, Icks A  Silke Kuske  Heinrich-Heine-University  Faculty of Medicine  Institute for Health Services Research and Health Economics  Moorenstraße 5  40225 Düsseldorf  silke.kuske@ddz.uni-duesseldorf.de  German Diabetes Center (DDZ)  Leibniz Institute for Diabetes Research  at Heinrich Heine University Düsseldorf  Paul-Langerhans-Group for Health Services Research and Health Economics  Auf'm Hennekamp 65  40225 Düsseldorf, Germany  Fliedner Fachhochschule  University of Applied Sciences  Geschwister-Aufricht-Straße 9  40489 Düsseldorf  Kai Moschinski  Heinrich Heine University  Faculty of Medicine  Institute for Health Services Research and Health Economics  Moorenstraße 5  40225 Düsseldorf  kai.moschinski@googlemail.com  Silke Andrich  Heinrich-Heine-University  Faculty of Medicine  Institute for Health Services Research and Health Economics  Moorenstraße 5  40225 Düsseldorf  silke.andrich@ddz.uni-duesseldorf.de  German Diabetes Center (DDZ)  Leibniz Institute for Diabetes Research  at Heinrich Heine University Düsseldorf  Institute for Biometrics and Epidemiology  Auf'm Hennekamp 65  40225 Düsseldorf, Germany  German Diabetes Center (DDZ)  Leibniz Institute for Diabetes Research  at Heinrich Heine University Düsseldorf  Paul-Langerhans-Group for Health Services Research and Health Economics  Auf'm Hennekamp 65  40225 Düsseldorf, Germany  Astrid Stephan  Heinrich Heine University  Faculty of Medicine  Institute for Health Services Research and Health Economics  Moorenstraße 5  40225 Düsseldorf  astrid.stephan@uni-duesseldorf.de  RWTH Aachen University Hospital  Department of Nursing  52074 Aachen  Irmela Gnass  Heinrich-Heine-University  Faculty of Medicine  Institute for Health Services Research and Health Economics  Moorenstraße 5  40225 Düsseldorf, Germany  irmela.gnass@uni-duesseldorf.de  Erika Sirsch  Facutly of nursing science  Vallendar Philosophical-Theological College  Palottistraße 3  56179 Vallendar, Germany  esirsch@pthv.de  Andrea Icks  Heinrich Heine University  Faculty of Medicine  Institute for Health Services Research and Health Economics  Centre for Health and Society  Moorenstraße 5  40225 Düsseldorf  andrea.icks@uni-duesseldorf.de  German Diabetes Center (DDZ)  Leibniz Institute for Diabetes Research  at Heinrich Heine University Düsseldorf  Paul-Langerhans-Group for Health Services Research and Health Economics  Auf'm Hennekamp 65  40225 Düsseldorf, Germany  German Center for Diabetes Research (DZD)  Ingolstädter Landstraße 1  85764 Neuherberg, Germany | 3a | Provide name, institutional affiliation, e-mail address of all protocol authors; provide physical mailing address of corresponding author |

| **Contributions:**  SK, SA, AS, AI and KM contributed to the concept. KM, SK, IG, and ES participated in the development of the systematic search strategies. SK and KM made contributions to the design, write-up and editing of the protocol. All authors revised critically and approved the final manuscript of the protocol. | 3b | Describe contributions of protocol authors and identify the guarantor of the review |
| --- | --- | --- |
| **Amendments**:  If we need to amend this protocol, we will give the date of each amendment, describe the change and give the rationale in this section. Changes will not be incorporated into the protocol. | 4 | If the protocol represents an amendment of a previously completed or published protocol, identify as such and list changes; otherwise, state plan for documenting important protocol amendments |
| **Support**: |  |  |
| This Protocol has received no funding. | 5a | Indicate sources of financial or other support for the review |
| Heinrich-Heine University is the sponsor for this review. | 5b | Provide name for the review funder and/or sponsor |
|  | 5c | Describe roles of funder(s), sponsor(s), and/or institution(s), if any, in developing the protocol |
| INTRODUCTION | | |
| **Rationale:**  Studies show that people with cognitive impairment, e.g. people with dementia, do not receive the same amount of analgesia after a hip- or pelvic fracture when compared to people without cognitive impairment | 6 | Describe the rationale for the review in the context of what is already known |
| **Objectives**:  The aim of this systematic review is to identify studies addressing drug-based pain management for people with dementia who have had a hip or pelvic fracture for which they had either an operation or conservative treatment. We will analyse to what extent and how the drug-based pain treatment for people with dementia is performed across all settings and how it is assessed in the studies. | 7 | Provide an explicit statement of the question(s) the review will address with reference to participants, interventions, comparators, and outcomes (PICO) |
| METHODS | | |
| **Eligibility criteria:**  Literature addressing drug-based pain management for PwD following hip or pelvic fractures in all settings will be included. Original articles reporting qualitative, quantitative or mixed methods studies and grey literature, such as dissertations, will be included. Letters, short reports, abstracts, editorials, comments or discussion papers will be screened in order to identify further original studies. We will include publications in which our topic has a primary or secondary focus. Publications without available references will be excluded. | 8 | Specify the study characteristics (such as PICO, study design, setting, time frame) and report characteristics (such as years considered, language, publication status) to be used as criteria for eligibility for the review |
| **Information sources:**  MEDLINE, EMBASE, CINAHL, Web of Knowledge and ScienceDirect will be searched. Backward citation tracking and forward citation tracking of selected literature will be added [1]. | 9 | Describe all intended information sources (such as electronic databases, contact with study authors, trial registers or other grey literature sources) with planned dates of coverage |
| **Search strategy:**  The search strategy will be set up by using the database-specific vocabularies (MeSH, EMTREE) and additional free text terms. The preliminary search algorithm is shown in Appendix I and II. The aim is to identify all relevant publications and at the same time to identify only those publications that are relevant (i.e. high sensitivity, low specificity), thus yielding a low Number-Needed-to-Read (NNR) and minimizing the subsequent workload. The search algorithm will be counter-checked by experienced reviewers and then piloted. Included search terms will be, for example, “analgesia”, “dementia”, “cognitive impairment”, “pain treatment”, “hip fracture” or “pelvic fracture”. | 10 | Present draft of search strategy to be used for at least one electronic database, including planned limits, such that it could be repeated |
| **Study records:** |  |  |
| Data management:  Literature search results will be uploaded to Rayyan, an Internet based software program that facilitates collaboration among reviewers. Furthermore, Endnote will be used. | 11a | Describe the mechanism(s) that will be used to manage records and data throughout the review |
| Selection process:  Inclusion and exclusion criteria will be pre-tested by conducting a comprehensive pre-screening and by discussing first core publications. Articles will be selected by title and by screening abstracts and will be checked by all the reviewers independently. The full texts selected will be double-checked. Unclear decisions will be resolved by consulting an additional reviewer.  Inter-rater reliability will be determined following title and abstract screening as well as after reviewing the full texts. | 11b | State the process that will be used for selecting studies (such as two independent reviewers) through each phase of the review (that is, screening, eligibility and inclusion in meta-analysis) |
| Data collection process:  A data extraction sheet will be developed according to Cochrane requirements. After data extraction, a content analysis will be conducted by developing categories (deductively and inductively) according to the topics of the review questions. We will also perform a descriptive quantitative analysis, taking for example prevalences or odds ratios into consideration. The analysis and synthesis of analysing differences and similarities between the studies will be performed as a peer group process. | 11c | Describe planned method of extracting data from reports (such as piloting forms, done independently, in duplicate), any processes for obtaining and confirming data from investigators |
| Data items:  First, an overview of the studies will be set up, stating author, year of publication, study design, settings, study objectives, findings regarding the extent of and methods used in the drug-based pain treatment of PwD in all settings, and the results of the critical appraisal. Second, data will be extracted and analysed with regard to the study methods (e.g. data collection by chart review or questionnaire), outcomes measures, type of data (e.g. clinical or administrative data), sample size, comparison between PwD and cognitively intact people, participant subgroups, and predictors. Third, data will be extracted and analysed that addresses the assessments of the studies identified, e.g. mental tests, pain scales, and type of medication, in line with the World Health Organization classification. A coding protocol will be used. | 12 | List and define all variables for which data will be sought (such as PICO items, funding sources), any pre-planned data assumptions and simplifications |
| **Outcomes and prioritization**  The primary outcome will be drug based pain management for people with dementia after hip or pelvic fractures across all settings in comparison with people without cognitive impairment.  The secondary outcome will be the consequences of insufficient drug based pain management for people with dementia after hip or pelvic fractures across all settings. | 13 | List and define all outcomes for which data will be sought, including prioritization of main and additional outcomes, with rationale |
| **Risk of bias in individual studies:**  All instruments that will be used to critically evaluate the selected studies will consider the risk of bias. To improve the value each study type will be assessed separately. | 14 | Describe anticipated methods for assessing risk of bias of individual studies, including whether this will be done at the outcome or study level, or both; state how this information will be used in data synthesis |
| **Data synthesis**  We do not perform a Meta-Analysis.  We will perform a descriptive qualitative analysis and quantitative analysis, taking for example prevalences or odds ratios into consideration. | 15a | Describe criteria under which study data will be quantitatively synthesised |
|  | 15b | If data are appropriate for quantitative synthesis, describe planned summary measures, methods of handling data and methods of combining data from studies, including any planned exploration of consistency (such as I^2^, Kendall’s τ) |
|  | 15c | Describe any proposed additional analyses (such as sensitivity or subgroup analyses, meta-regression) |
|  | 15d | If quantitative synthesis is not appropriate, describe the type of summary planned |
| **Meta-bias(es)**  A critical appraisal will be performed to identify bias. | 16 | Specify any planned assessment of meta-bias(es) (such as publication bias across studies, selective reporting within studies) |
| **Confidence in cumulative evidence:**  The studies will be checked by using the instruments of the Scottish Intercollegiate Guideline Networks (SIGN). These were chosen due to the evaluation of different grading systems by Baker et al. They pointed out, that the SIGN-instruments are easy to handle and that they offer a broad spectrum of Checklists for the different study types. Furthermore, SIGN features clear criteria, questions that are easy to understand and provide solid results. Studies that are not addressed in the SIGN guideline will be analyzed using the critical appraisal tools of the National Institute for Health and Care Excellence (NICE). Mixed Method studies, which are addressed neither by the SIGN nor by the NICE guidelines will be accessed by the Mixed Methods Appraisal Tool (MMAT) – Version 2011. This process will be performed independently by two reviewers | 17 | Describe how the strength of the body of evidence will be assessed (such as GRADE) |

*** It is strongly recommended that this checklist be read in conjunction with the PRISMA-P Explanation and Elaboration (cite when available) for important clarification on the items. Amendments to a review protocol should be tracked and dated. The copyright for PRISMA-P (including checklist) is held by the PRISMA-P Group and is distributed under a Creative Commons Attribution Licence 4.0.**

*From: Shamseer L, Moher D, Clarke M, Ghersi D, Liberati A, Petticrew M, Shekelle P, Stewart L, PRISMA-P Group. Preferred reporting items for systematic review and meta-analysis protocols (PRISMA-P) 2015: elaboration and explanation. BMJ. 2015 Jan 2;349(jan02 1):g7647.*
